# The Functions of PCNA in Tumor Stemness and Invasion

**DOI:** 10.3390/ijms23105679

**Published:** 2022-05-19

**Authors:** Yuan-Liang Wang, Wan-Rong Wu, Pei-Le Lin, Yi-Chun Shen, You-Zhe Lin, Hong-Wei Li, Kai-Wen Hsu, Shao-Chun Wang

**Affiliations:** 1 Graduate Institute of Biomedical Sciences, College of Medicine, China Medical University, Taichung 40402, Taiwan; yuanliang0402@gmail.com (Y.-L.W.); happy9827000@gmail.com (W.-R.W.); rebecca19920927@gmail.com (P.-L.L.); u104076012@cmu.edu.tw (Y.-C.S.); yaweyoung@gmail.com (Y.-Z.L.); 2 Center for Molecular Medicine, China Medical University Hospital, Taichung 40447, Taiwan; 3 Research Center for Cancer Biology, China Medical University, Taichung 40402, Taiwan; albertli489@gmail.com (H.-W.L.); kwhsu@mail.cmu.edu.tw (K.-W.H.); 4 Institute of Translational Medicine and New Drug Development, China Medical University, Taichung 40402, Taiwan; 5 Drug Development Center, China Medical University, Taichung 40402, Taiwan; 6 Cancer Biology and Drug Discovery Ph.D. Program, China Medical University, Taichung 40402, Taiwan; 7 Department of Biotechnology, Asia University, Taichung 41354, Taiwan; 8 Department of Cancer Biology, University of Cincinnati, Cincinnati, OH 45267, USA

**Keywords:** PCNA, phosphorylation, invasion, stromal activity, stemness, tumor-initiating cells, mammosphere, ALDH, breast cancer

## Abstract

Invasion is the most prominent lethal feature of malignant cancer. However, how cell proliferation, another important feature of tumor development, is integrated with tumor invasion and the subsequent cell dissemination from primary tumors is not well understood. Proliferating cell nuclear antigen (PCNA) is essential for DNA replication in cancer cells. Loss of phosphorylation at tyrosine 211 (Y211) in PCNA (pY211-PCNA) mitigates PCNA function in proliferation, triggers replication fork arrest/collapse, which in turn sets off an anti-tumor inflammatory response, and suppresses distant metastasis. Here, we show that pY211-PCNA is important in stromal activation in tumor tissues. Loss of the phosphorylation resulted in reduced expression of mesenchymal proteins as well as tumor progenitor markers, and of the ability of invasion. Spontaneous mammary tumors that developed in mice lacking Y211 phosphorylation contained fewer tumor-initiating cells compared to tumors in wild-type mice. Our study demonstrates a novel function of PCNA as an essential factor for maintaining cancer stemness through Y211 phosphorylation.

## 1. Introduction

Invasion of cancer cells is the hallmark and the most life-threatening characteristic of malignant tumors [1,2]. An immense body of knowledge has been accumulated in the past decade on how the invasive phenotype, often featured by stromal activation and epithelial–mesenchymal transition (EMT) in the tumor microenvironment, is regulated in cancerous cells to foster lethal metastasis [3]. Accompanying the enhanced metastatic potential is the promoted programming for cancer stemness, manifested by the increased activity of the stem-like tumor initiating cells [4]. It has been demonstrated that the expression of the EMT-driving genes also drives the stemness phenotype of cancer stem cells [4]. Conversely, cancer stem cells, as defined on the basis of the phenotype of tumor initiation and lineage differentiation, constitute the main taskforce of distant metastasis from primary tumors [5].

Active cell proliferation as a universal feature of malignant cells plays an important function in tumor development and progression [1]. It is intuitive to speculate that regulation of cell proliferation is part of stem cell biology in which sustained self-renewal is one of the key features [6]. However, whether the cell proliferation machinery participates in programming the stemness of tumor-initiating cells and whether this can lead to cancer invasion and metastasis remains largely unaddressed.

For the replication of the entire genome, PCNA forms a trimeric ring encircling the DNA double helix and functioning as the scaffolding protein of the DNA synthesis machinery at the replication forks [7]. Thus, PCNA is indispensable for cell proliferation and survival, whereas deficiency of the *Pcna* gene results in embryonic lethality in mice [8]. PCNA also maintains genomic integrity through its axillary functions in DNA synthesis to repair DNA damages through double-strand breaks repair, mismatch repair, base excision repair, and nucleotide excision repair [7,9]. In addition to DNA synthesis and repair, following DNA replication, PCNA recruits a protein complex to reassemble the chromatin of the post-replication DNA double helix and replace pre-replication epigenetic memories [10].

Studies in the past decades have established that the functions of PCNA are regulated through post-translational modifications [7,9,11]. Among them, Y211 phosphorylation in PCNA preferentially promotes proliferation of cancer cells [12,13,14,15,16]. Mutation of the Y211 residue has been documented in human cancer [17]. We recently reported the engineering of knock-in mice in which the wild-type *Pcna* gene was replaced with the 211F mutant in which the tyrosine 211 residue was substituted with a phenylalanine residue. The resulting mutant mice, namely, the 211F/PCNA strain, developed normally compared to wild-type mice. The two strains were then crossed with the MMTV-PyMT transgenic line that carries the oncogene polyoma middle-T antigen driven by the mouse mammary tumor virus promoter/enhancer. The resulting mouse strains, WT/PyMT and 211F/PyMT, respectively, develop spontaneous tumors in the mammary glands. Consistent with the known proliferating function of Y211 phosphorylation [12,13,14,15,16], tumors in WT/PyMT mice grew faster than in 211F/PyMT mice [18]. Moreover, it was demonstrated that loss of phosphorylation at tyrosine 211 (Y211) of PCNA resulted in deficiency of DNA metabolism, leading to the presence of single-stranded DNA in the cytoplasm, which in turn triggered the cGAS–STING stress signaling pathway, resulting in inflammatory response and anti-tumor immunity, thus preventing distant metastasis [18].

These results demonstrated a link between cell proliferation and the programming of the tumor immune microenvironment through Y211 phosphorylation in PCNA. The current study addresses whether this signaling event also participates in promoting tumor initiation and unleashes the invasiveness of cancer cells.

## 2. Results

### 2.1. Enhanced Mesenchymal Phenotype in Spontaneous Tumors Derived from WT/PyMT Mice but Not in Tumors Developed in 211F/PyMT Mice

**Figure 1:**
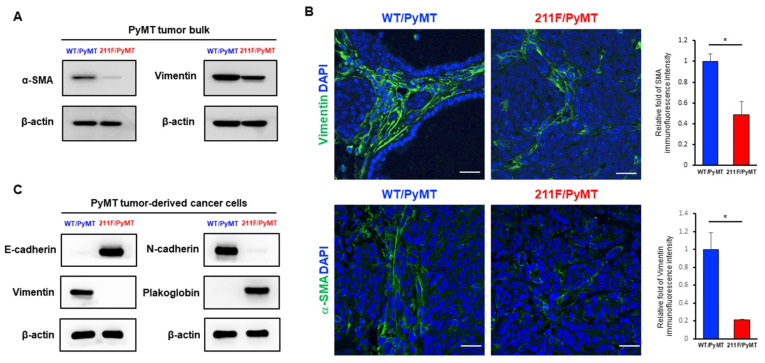
Enhanced mesenchymal phenotype in spontaneous tumors derived from WT/PyMT mice but not in tumors developed in 211F/PyMT mice. (**A**), Protein extracts were prepared from the tumors isolated from 10-week-old MMTV/PyMT mice of the indicated strains; expression of vimentin, SMA and the internal control β-actin was characterized by immunoblotting. (**B**), Immunofluorescence staining of vimentin and α-SMA in PyMT tumor tissues of the indicated strains. Representative images of immunofluorescence staining are shown. Bar, 25 μm. Vimentin and α-SMA expression in the PyMT cells was quantitated by ImageJ. The relative fold changes were calculated by the staining ratios of FITC to DAPI. Bar, standard deviation. *, *p* < 0.05 by Student’s *t*-test. (**C**), Western blotting analysis of the indicated EMT markers in WT and Y211F PyMT tumor-derived cells.

**Figure 2:**
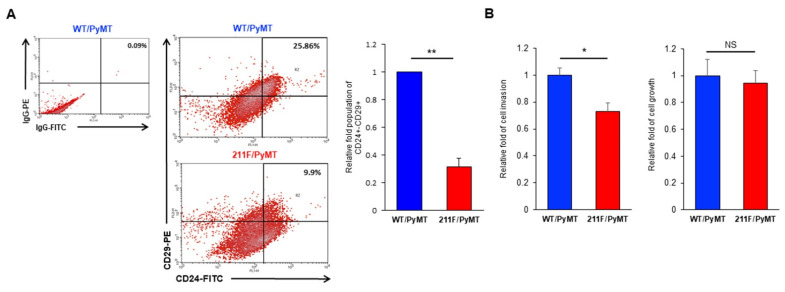
Increased stemness markers in WT/PyMT tumors. (**A**), Expression of the cancer stemness markers CD24 and CD29 in PyMT-tumor derived cells was determined by flow cytometry. Quantification results and representative plots are shown. The isotype control IgG antibodies were employed to exclude non-specific staining. Bar, standard deviation. **, *p* < 0.01 by Student’s *t*-test. (**B**), Cell invasion and migration were quantitated and showed that the WT/PyMT cells had higher invasion ability but no difference in cell growth compared to the 211F/PyMT cells. Bar, standard deviation. *, *p* < 0.05 by Student’s *t*-test. NS, non-significance.

We recently reported the production of an homozygous strain with both alleles of PCNA replaced with the 211F mutant in a spontaneous oncogenic background of MMTV-PyMT [18]. The WT/PyMT tumors also showed significant ability of lung metastasis formation compared to the 211F/PyMT tumors. Indeed, examining the protein extract of the tumor tissues, we found that that the WT/PyMT tumors expressed increased levels of the smooth muscle α (α-SMA) protein and vimentin compared to the 211F/PyMT tumors (Figure 1A). This was further confirmed by immunofluorescent staining of the stromal phenotype, which suggested an enhanced mesenchymal activity (Figure 1B) [19]. Consistently, Western blotting analysis demonstrated that the PyMT tumor-derived cancer cells expressed drastically higher levels of stromal proteins (Figure 1C). We speculated that the enhanced stromal activity was associated with increased stemness. To test this hypothesis, cells isolated from the primary tumors were stained for the surface proteins CD24 and CD29 and analyzed by fluorescence-activated cell sorting (FACS). Co-expression of CD24 and CD29 is a signature of mammary stem cells [20]. Indeed, the WT/PyMT tumors contained a higher percentage of CD24^+^CD29^+^ cells compared to the 211F/PyMT tumors (Figure 2A), suggesting that the WT/PyMT tumors contained more stem cell-like cells than the 211F/PyMT tumors and predicting higher invasiveness of the WT/PyMT-derived cancer cells than of the 211F/PyMT cells. To test this possibility, Boyd chamber invasion assays were conducted, which showed that the WT/PyMT cells exhibited increased invasiveness, but not cell growth, compared to the 211F/PyMT cells (Figure 2B). These results are in line with the fact that enhanced epithelial mesenchymal transition is often accompanied by increased cancer stemness [4,5].

### 2.2. Y211 Phosphorylation Is Important for Maintaining the Stemness of Cancer Cells

**Figure 3:**
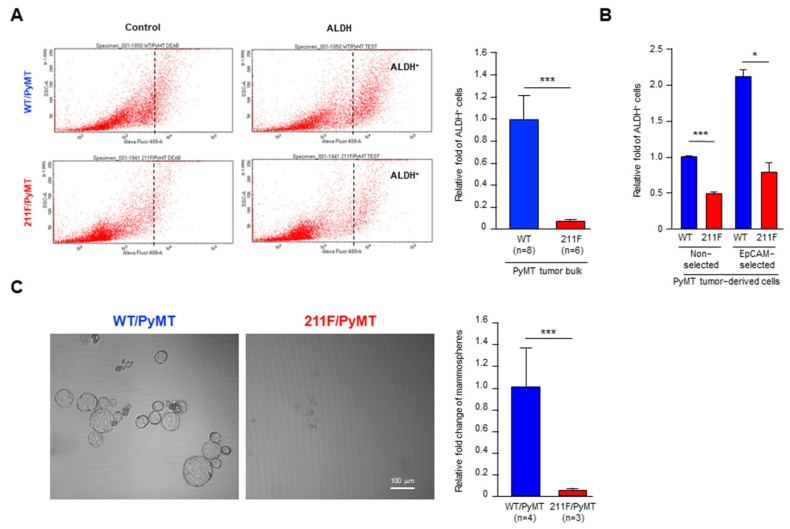
Y211 phosphorylation is important for maintaining the stemness of cancer cells in vitro. (**A**), Left, representative plots of the ALDH^+^ cell sub-population isolated from WT/PyMT and 211F/PyMT tumor tissues. Right, data from multiple tumors of each genotype were quantitated and plotted. The numbers of mice are indicated (n). (**B**), Bulk cell populations were independently isolated from a pair of WT/PyMT and 211F/PyMT tumor tissues, and their ALDH activities were compared either in the straight cell population (non-selected) or in the cell population after enrichment for epithelial cells by EpCAM pull-down (EpCAM−selected). The data derived from three independent pairs of tumor tissues were quantitated and plotted. Bar, standard deviation. *, *p* < 0.05, ***, *p* < 0.005 as determined by Student’s *t*-test. (**C)**, Left, representative images of mammosphere formation by WT/PyMT and 211F/PyMT cells. Right, result quantification showed that mammosphere formation was compromised in 211F/PyMT. ***, *p* < 0.005 as determined by Student’s *t*-test.

To further interrogate the role of Y211 phosphorylation in tumor initiation, we first measured the enzyme activity of aldehyde dehydrogenase (ALDH)—a generally accepted stem cell marker of the mammary glands and correlated with poor prognosis in breast cancer [21]—in cancer cells isolated from the PyMT tumors. Cancer cells isolated from the WT/PyMT tumors harbored about 10 times more ALDH activity than cells isolated from the 211F/PyMT tumors (Figure 3A,B). The increase of ALDH activity predicted a promoted ability of tumor initiation and maintenance, which can be assessed by the cell ability of forming mammospheres in suspension culture [22,23]. The WT/PyMT cancer cells showed a prominent ability to develop tumorspheres compared to the 211F/PyMT cells (Figure 3C). Together, the results of the biochemical and cellular assays support the conclusion that the cancer cells derived from the WT/PyMT tumors contained higher tumor-initiating activity than the cells derived from the 211F/PyMT tumors.

**Figure 4:**
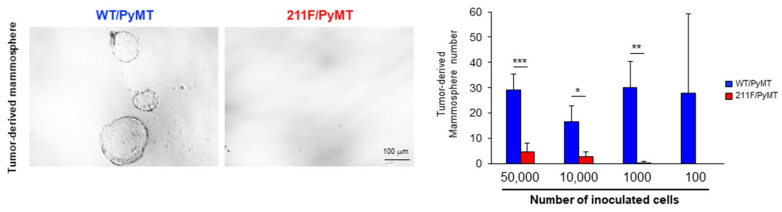
Y211 phosphorylation is important for maintaining the stemness of cancer cells in vivo. (**Left**), representative mammosphere formation is shown. (**Right**), tumor cells isolated from limiting dilution-derived tumor tissues were inoculated in suspension culture, and the formed mammospheres were counted. Bar, standard deviation. *, *p* < 0.05; **, *p* < 0.01; ***, *p* < 0.001 as determined by Student’s *t*-test.

The ability of tumor regrowth as assessed by transplanting limiting dilutions of cancer cells derived from spontaneous tumors is the gold standard characterizing tumor initiating activity [20]. Accordingly, different numbers of epithelial cancer cells isolated from the PyMT tumors of each genotype were orthotopically transplanted in the mammary glands of immune-deficient mice. Based on the assays, the estimated stem cell frequency of the WT/PyMT tumor cells was 1/17228, significantly higher than the estimated frequency of 1/62899 of the 211F/PyMT cells (Table 1). The tumors derived from the implanted PyMT cells were excised, and tumor cells were isolated to be tested for their ability of tumorsphere formation. Consistent with the inheritable characteristic of stemness, cells isolated from the WT/PyMT tumors maintained a relatively higher tumorsphere forming ability compared to cells isolated from the 211F/PyMT tumors (Figure 4). Together, these results showed that the WT/PyMT cancer cells harbored a higher tumor initiation activity compared to the 211F/PyMT cells, supporting a function for Y211 phosphorylation in PCNA in cell stemness during tumorigenesis.

### 2.3. Metabolism Reprogramming Mediated by Y211 Phosphorylation in PCNA

**Figure 5:**
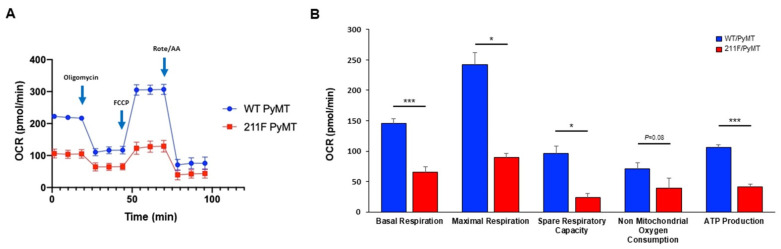
Mitochondrial activities regulated by PCNA Y211 phosphorylation. (**A**), OCR was determined by the Agilent Seahorse XFe24 Extracellular Flux analyzer for the PyMT tumor−derived cells of the indicated genotypes in the presence of the indicated metabolic inhibitors to determine basal, maximal, spare respiratory activities, as well as non-mitochondrial oxygen consumption and ATP production. (**B**), OCR measurements. Bar, standard deviation. *, *p* < 0.05; ***, *p* < 0.005 as determined by Student’s *t*-test.

Mitochondria are the powerhouses of cells, being the main source of ATP generation, especially in slowly proliferating cells or well-differentiated somatic cells. Cancer cells are highly proliferating cells which rely on rapid ATP production and on metabolic intermediates as building blocks to support their growth and division through aerobic glycolysis. It has been shown that oxidative phosphorylation (OXPHOS) through mitochondrial respiration is the major energy source and a phenotypic hallmark associated with cancer stemness [24,25]. To assess the mitochondrial function in relation to Y211 phosphorylation, the mitochondrial oxygen consumption rate (OCR) was measured by the Seahorse extracellular flux assay (Figure 5A). Three metabolic compound inhibitors including the complex V inhibitor oligomycin, the ionophore carbonyl cyanide-4-(trifluoromethoxy) phenylhydrazone (FCCP), and the combination of complex I and III inhibitors, rotenone and antimycin A, were sequentially used in this assay. Compared to 211F/PyMT cells, WT/PyMT cells revealed a significant increase in maximal respiration, spare respiratory capacity, and ATP production, indicating that Y211 phosphorylation promotes OXPHOS, which is associated with the stem cell phenotype (Figure 5B).

### 2.4. MEFs Derived from WT Mice (MFE/WT) Promote Cancer Cell Growth Compared to MEFs Derived from 211F Mice (MEF/211F)

**Figure 6:**
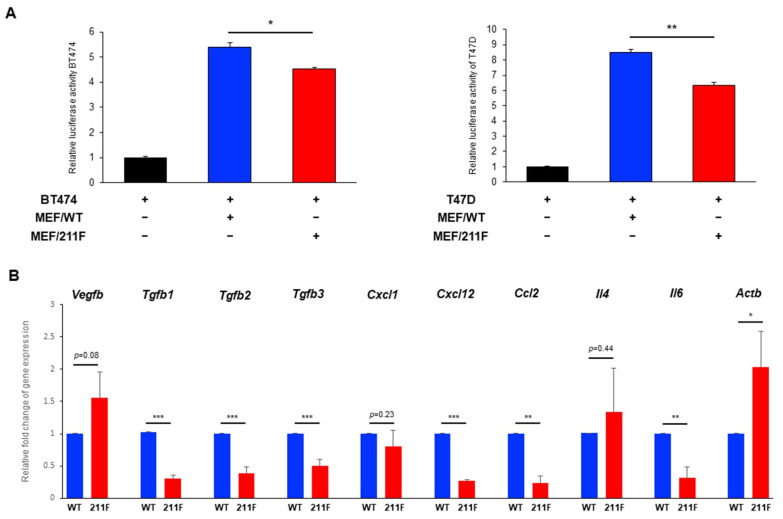
MEFs derived from WT mice (MFE/WT) promote cancer cell growth compared to MEFs derived from 211F mice (MEF/211F). (**A**), MEFs were co-cultured with human breast cancer cells stably expressing the luciferase reporter, BT474−luc and T47D−luc, at a 1:1 ratio for 48 h. Luciferase activity was measured by a luminometer. Bar, standard deviation. *, *p* <0.05; **, *p* < 0.01 as determined by Student’s *t*-test. (**B**), RNA expression of cytokines and chemokines in WT and Y211F MEF cells was determined by quantitative real-time PCR. which was normalized by the expression of the *Rn18s* reference gene. * *p* < 0.05, ** *p* < 0.01, *** *p* < 0.005.

As well as the invasive activity, the enhanced stromal activity in WT/PyMT tumors compared to the 211F/PyMT tumors also suggested that the stromal compartment in the WT/PyMT background can facilitate cancer cell growth. To test this possibility, human breast cancer cell lines were tested for their growth when co-cultured with mouse embryonic fibroblasts (MEFs) from WT/WT and 211F/211F mice. To facilitate the evaluation of cell growth, the cancer cells BT474 and T47D were transiently transfected with a luciferase reporter gene driven by the constitutive cytomegalovirus (CMV) promoter before culturing them with or without MEFs. After three days of incubation, the cultures were lysed, and luciferase activity was measured by a luminometer (Figure 6A). The results showed that whereas incubation with MEFs of either genotype significantly enhanced the growth of both lines, WT MEFs had stronger growth-promoting activity than 211F MEFs. To further investigate the potential crosstalk between cancer cells and fibroblasts in relation to Y211 phosphorylation, the expression of a number of known tumor stroma-associated molecules was assessed by quantitative reverse-transcription polymerase chain reaction (qRT-PCR). Numerous cytokines and chemokines known to enhance stromal activity such as TGFB1, TGFB2, TGFB3, CXCL12, CCL2 and IL-6 were found downregulated in 211F MEF cells, suggesting a potential tumor-promoting function of Y211 phosphorylation through regulation of the secretome in the stromal environment (Figure 6B).

## 3. Discussion

Together with prior studies, WT/PyMT cells, compared to 211F/PyMT cells, exhibit pro-tumor advantages in multiple cellular functions including cell proliferation [11,12], modulation of the immune microenvironment [18], and, as supported by the current studies, stromal activation as well as enhancement of cancer stemness. Collectively, these results are consistent with a notable role for Y211 phosphorylation in PCNA in maintaining the stemness of cancer cells and tumor invasiveness.

Peng et al. reported recently that the role of Y211 phosphorylation in PCNA was tested by generating a HeLa cell-derived cell line in which WT, 211F, and the potentially phosphomimetic mutant 211D (aspartic acid) of the *PCNA* transgene was ectopically inserted into the *AAVS1* intron area by CRISPR/Cas9 [26]. The study further showed that the 211D PCNA conveyed elevated levels of EMT in vitro via the ATM/Akt/GSK3β/Snail signaling pathway. Thus, both studies by Peng et al. and by our group drew similar conclusions, supporting the idea that Y211 phosphorylation in PCNA is important for cancer invasion. The current study further pinpoints the role of Y211 phosphorylation in activating stromal activity in spontaneous tumors and in maintaining tumor initiation activity both in cell culture and in animal models. Our most recent report showed that Y211 phosphorylation in PCNA is critical for the integrity of genomic DNA, and the loss of Y211 phosphorylation results in aberrant generation of ssDNA in the cytosol. Consistently, the loss of Y211 phosphorylation induces the cGAS-STING cascade, leading to an anti-tumor inflammatory response [18]. Collectively, these results illustrate that phosphorylation of PCNA at Y211 conveys versatile functions to promote tumor progression through distinct mechanisms.

Our study also raises a caveat regarding the utilization of phosphorylation-mimicking mutants such as 211E or 211D to study the physiological functions of PCNA Y211 phosphorylation. We generated genuine knock-in mice in which the endogenous WT *Pcna* gene was replaced by the 211E mutant. Albeit extensive attempts, we were not able to generate the homozygous strain of 211E PCNA, indicating a dose effect of phosphorylation in relation to cell viability, at least in the embryonic development stage. Further study is required to fully understand the impacts of this signaling event in cellular functions.

We showed increased expression of the mesenchymal molecules vimentin and αSMA in WT/PyMT tumors compared to the 211F/PyMT counterpart. Vimentin and αSMA in tumor tissues can be expressed by cancer cells, presumably through EMT, or by resident stromal cells such as fibroblasts. Our results demonstrated that the wild-type PyMT cancer cells expressed dramatically higher levels of the mesenchymal molecules than the 211F cells, supporting the essential function of Y211 phosphorylation in PCNA in driving a desmoplastic environment to facilitate invasion. It remains to be determined whether the phosphorylation event directly influences gene expression in cancer cells and whether cancer-associated stromal cells contribute to this gene expression in cancer cells. We reported that the presence and absence of Y211 phosphorylation may influence the ability of cancer cells to help shape the microenvironmental landscape [18]. The current results that the WT/PyMT tumors showed higher stromal in and local invasiveness than the 211F/PyMT tumors suggest that Y211 phosphorylation of PCNA plays an active role in shaping the tumor microenvironment for tumor progression in addition to its traditional growth-promoting function. Together, these results demonstrate that the phosphorylation of PCNA at Y211 is an example of how the regulation of the DNA replication core machinery on one hand is a signaling event influencing intrinsic cell growth functions including the maintenance of stemness, while on the other hand can also help shape the tumor microenvironment, which reprograms stromal activity, invasion and distant metastasis.

It remains to be determined whether phosphorylation of Y211 actively induces EMT in cancer cells, hence enhancing the expression of mesenchymal molecules such as αSMA and vimentin. Alternatively, pY211-PCNA can mediate or coordinate with a robust recruitment of stromal cells which orchestrate a desmoplastic microenvironment for tumor promotion. Thirdly, pY211-PCNA may also function in stromal cells to promote tumor growth. The latter conclusion is supported by the observation that co-culturing human breast cancer cells with WT MEF resulted in higher growth activity than co-culturing with 211F MEFs. The paracrine cytokines secreted by cancer-associated fibroblasts, such as TGFβ, CXCL12, CCL2 and IL-6, have been identified as important signaling molecules for tumor progression and immunosuppression in the microenvironment [27,28,29]. Interestingly, gene expression analysis showed that the expression of these cytokines in 211F MEFs was reduced compared to wild-type MEFs, suggesting that Y211 phosphorylation in PCNA may promote a cellular context regulating the secretion of cytokines, chemokines or growth factors. Together, the current results suggest that Y211 phosphorylation in PCNA plays a key role in the crosstalk between cancer cells and stroma to foster a conducive environment for tumor invasion. This finding provides a workable platform to dissect the genuine tumor microenvironment, including cancer-associated fibroblasts, mesenchymal stromal cells, as well as immune cells, to further understand the underlying mechanisms connecting cell proliferation and cancer metastasis.

## 4. Materials and Methods

### 4.1. Cell Lines, Chemicals and Antibodies

WT/PyMT and 211F/PyMT cells were generated as described previously [18]. PyMT-derived cells, T47D and BT474 cells were cultured in DMEM/F12 with 10% FBS and 1% penicillin–streptomycin (Thermo Fisher Scientific, Waltham, MA, USA). Anti-SMA and anti-plakoglobin antibodies were purchased from Abcam (Cambridge, UK); anti-vimentin and anti-actin antibodies were purchased from Santa Cruz (Dallas, TX, USA). Anti-E-cadherin and anti-N-cadherin antibodies were purchased from GeneTex (Irvine, CA, USA). Anti-CD24-FITC and CD29-PE antibodies for flow cytometry were purchased from Biolegend (San Diego, CA, USA). Mouse CD326 MicroBeads were purchased from Miltenyi Biotec (Bergisch Gladbach, Germany). Collagenase type IV was purchased from Sigma-Aldrich (St. Louis, MO, USA).

### 4.2. Boyden Chamber Assay

Matrigel (Corning, Corning, NY, USA) was applied to Transwell inserts (Corning, Corning, NY, USA) the day prior to plating cells in them. Cells were suspended in serum-free medium and plated into the upper chamber, while 500 μL of complete medium was placed in the lower chamber. Cells were incubated for 72 h, and cell invasion was quantitated by crystal violet staining.

### 4.3. Immunofluorescence

Cells were plated on glass coverslips overnight. Fixation was performed in 4% paraformaldehyde at room temperature for 15 min. For permeabilization, cells were incubated in 0.25% Triton-X100/PBS at room temperature for 10 min. The fixed cells were then blocked in 5% goat serum in PBS for 1 h, followed by staining with primary antibodies at 4 °C overnight, washing, and staining with fluorophore-conjugated secondary antibodies at room temperature for 1 h. Slides were mounted and examined under a confocal microscope.

### 4.4. Limiting Dilution Assay

PyMT tumors were minced and homogenized in C tubes with a Gentle MACS (Miltenyi Biotec, Bergisch Gladbach, Germany). The tissues were digested in the digestion medium containing 1 mg/mL Collagenase IV, 2.5% FBS, 10 mM HEPES pH7.5, and 1% Pen–Strep in RPMI 1640. Cells were collected by centrifugation, and the pellets were suspended in ACK lysis buffer (Thermo Fisher Scientific, Waltham, MA, USA ) at 37 °C for 5 min. The cell mixture was passed through a 70 μm cell strainer. CD326 (EpCAM)^+^ cells were enriched with CD326 microbeads and re-suspended in 50 μL of 50% Matrigel (Corning, Corning, NY, USA) in DMEM-F12. The cells were counted by a hemocytometer. The cells were then transplanted into the fat pads of six- to seven-week-old female NOD/SCID mice. The mice were monitored until palpable tumors developed. The result was analyzed using an online tool (http://bioinf.wehi.edu.au/software/elda/) (accessed on 26 June 2019).

### 4.5. ALDH Assay

Single-cell suspensions were prepared from PyMT tumors or PyMT cells, and ALDH activity was measured by following the manufacturer’s protocol (STEMCELL Technology, Vancouver, BC, Canada). Briefly, cell pellets were re-suspended in 1 mL of the assay buffer. Then, 5 μL of the Aldefluor reagent was added to the mixture with and without 5 μL of the ALDH inhibitor DEAB. The reactions were incubated at 37 °C for 30 min, and the enzyme activity was measured by FACS analysis. The reaction containing DEAB was set as the negative control.

### 4.6. Mammosphere Formation Assay

Cultured cells were trypsinized by 0.1% trypsin, and the cellularity was confirmed using a hemocytometer. The cells were inoculated in triplicates at the appropriate densities in 2 mL of mammosphere medium in 6-well ultralow-attachment plates. The plates were incubated in a humidified atmosphere at 37 °C with 5% CO_2_ for 5 days without disturbing them. The number of mammospheres was determined by counting under a microscope at 200× magnification. The mammosphere-forming efficiency was calculated by the formula: (number of mammospheres per well/ number of cells seeded per well) × 100.

### 4.7. Oxygen Consumption Rate (OCR) Assay

Mitochondrial respiratory activity was measured using the Seahorse XFe24 Extracellular Flux analyzer following the manufacturer’s instructions.

### 4.8. Fibroblast Co-Culture Assay

T47D and BT474 cells were transiently transfected with a CMV promoter-driven luciferase reporter by lipofectamine 2000, then co-cultured with MEFs at the ratio of 1 to 1 (10,000 cells for each) for 48 h. Luciferase activity measurements were performed following the manufacturer’s protocol (Promega, Madison, WI, USA).

### 4.9. qRT-PCR

Total RNA was isolated using TRIzol reagent (ThermoFisher Scientific, Waltham, MA, USA) and the Direct-zol RNA kit (Zymo Research, Irvine, CA, USA) according to the manufacturer’s protocol. cDNA synthesis was performed in M-MLV reverse transcriptase (Thermo Fisher Scientific, Waltham, MA, USA ). qRT-PCR was performed by using SYBR Green master mix (Bio-rad, Hercules, CA, USA) and a QuantStudio 5 real-time PCR system (Applied Biosystems, Waltham, MA, USA). Relative levels were calculated using the comparative CT method. The qRT-PCR primer sequences were acquired from the PrimerBank database [30]. *Vegfb* forward: 5′-GCCAGACAGGGTTGCCATAC-3′; *Vegfb* reverse: 5′-GGAGTGGGATGGATGATGTCAG-3′; *Tgfb1* forward: 5′-CTCCCGTGGCTTCTAGTGC-3′; *Tgfb1* reverse: 5′-GCCTTAGTTTGGACAGGATCTG-3′; *Tgfb2* forward: 5′-TCGACATGGATCAGTTTATGCG-3′; *Tgfb2* reverse: 5′-CCCTGGTACTGTTGTAGATGGA-3′; *Tgfb3* forward: 5′-CCTGGCCCTGCTGAACTTG-3′; *Tgfb3* reverse: 5′-TTGATGTGGCCGAAGTCCAAC-3′; *Cxcl1* forward: 5′-ACTGCACCCAAACCGAAGTC-3′; *Cxcl1* reverse: 5′-TGGGGACACCTTTTAGCATCTT-3′; *Cxcl12* forward: 5′-TGCATCAGTGACGGTAAACCA-3′; *Cxcl12* reverse: 5′-TTCTTCAGCCGTGCAACAATC-3′; *Ccl2* forward: 5′-TTAAAAACCTGGATCGGAACCAA-3′; *Ccl2* reverse: 5′-GCATTAGCTTCAGATTTACGGGT-3′; *Il4* forward: 5′-ACCTTGACGGTGTTCATACAGT-3′; Il4 reverse: 5′-CTGCTCCTATTCGACCACTATCT-3′; *Il6* forward: 5′-TAGTCCTTCCTACCCCAATTTCC-3′; *Il6* reverse: 5′-TTGGTCCTTAGCCACTCCTTC-3′; *Actb* forward: 5′-GGCTGTATTCCCCTCCATCG-3′; *Actb* reverse: 5′-CCAGTTGGTAACAATGCCATGT-3′.

## Figures and Tables

**Table 1:** Limiting dilution repopulation assay of WT/PyMT and 211F/PyMT cells.

			Confidence Intervals for Stem Cell Frequency			
Genotype	Cell ^#^	Tumor Incidence (Response/Test)	Estimate	Lower	Upper	*p*-Value
**WT/PyMT**	50,000 10,000 1000 100	14/16 8/14 2/16 0/18	1/17,228	28,660	10,356	0.000583
**211F/PyMT**	50,000 10,000 1000 100	10/16 1/16 0/18 0/18	1/62,899	113,621	34,820	

^#^ Cells isolated from the indicated PyMT tumors were transplanted into the NOD/SCID mice fat pads at cell numbers of 100, 1000, 10,000 and 50,000.

## Data Availability

Not applicable.

## References

[1] Hanahan D., Weinberg R.A. (2011). Hallmarks of Cancer: The Next Generation. Cell.

[2] Nguyen D.X., Bos P.D., Massagué J. (2009). Metastasis: From dissemination to organ-specific colonization. Nat. Rev. Cancer.

[3] Thiery J.P., Acloque H., Huang R.Y.J., Nieto M.A. (2009). Epithelial-Mesenchymal Transitions in Development and Disease. Cell.

[4] Mani S.A., Guo W., Liao M.-J., Eaton E.N., Ayyanan A., Zhou A.Y., Brooks M., Reinhard F., Zhang C.C., Shipitsin M. (2008). The Epithelial-Mesenchymal Transition Generates Cells with Properties of Stem Cells. Cell.

[5] Melo F.d.S.e., Kurtova A.V., Harnoss J.M., Kljavin N., Hoeck J.D., Hung J., Anderson J.E., Storm E.E., Modrusan Z., Koeppen H. (2017). A distinct role for Lgr5+ stem cells in primary and metastatic colon cancer. Nature.

[6] Blanpain C., Lowry W.E., Geoghegan A., Polak L., Fuchs E. (2004). Self-Renewal, Multipotency, and the Existence of Two Cell Populations within an Epithelial Stem Cell Niche. Cell.

[7] Moldovan G.L., Pfander B., Jentsch S. (2007). PCNA, the maestro of the replication fork. Cell.

[8] Roa S., Avdievich E., Peled J.U., MacCarthy T., Werling U., Kuang F.L., Kan R., Zhao C., Bergman A., Cohen P.E. (2008). Ubiquitylated PCNA plays a role in somatic hypermutation and class-switch recombination and is required for meiotic progression. Proc. Natl. Acad. Sci. USA.

[9] Choe K.N., Moldovan G.-L. (2017). Forging Ahead through Darkness: PCNA, Still the Principal Conductor at the Replication Fork. Mol. Cell.

[10] Maison C., Almouzni G. (2004). HP1 and the dynamics of heterochromatin maintenance. Nat. Rev. Mol. Cell Biol..

[11] Wang S.-C. (2014). PCNA: A silent housekeeper or a potential therapeutic target?. Trends Pharmacol. Sci..

[12] Wang S.-C., Nakajima Y., Yu Y.-L., Xia W., Chen C.-T., Yang C.-C., McIntush E.W., Li L.-Y., Hawke D.H., Kobayashi R. (2006). Tyrosine phosphorylation controls PCNA function through protein stability. Nat. Cell Biol..

[13] Zhao H., Lo Y.-H., Ma L., Waltz S.E., Gray J.K., Hung M.-C., Wang S.-C. (2011). Targeting tyrosine phosphorylation of PCNA inhibits prostate cancer growth. Mol. Cancer Ther..

[14] Lo Y.H., Ho P.C., Wang S.C. (2012). Epidermal growth factor receptor protects proliferating cell nuclear antigen from cullin 4A protein-mediated proteolysis. J. Biol. Chem..

[15] Zhao H., Ho P.-C., Lo Y.-H., Espejo A., Bedford M.T., Hung M.-C., Wang S.-C. (2012). Interaction of proliferation cell nuclear antigen (PCNA) with c-Abl in cell proliferation and response to DNA damages in breast cancer. PLoS ONE.

[16] Zhao H., Chen M.-S., Lo Y.-H., Waltz S., Wang J., Ho P.-C., Vasiliauskas J., Plattner R., Wang Y.-L., Wang S.-C. (2014). The Ron receptor tyrosine kinase activates c-Abl to promote cell proliferation through tyrosine phosphorylation of PCNA in breast cancer. Oncogene.

[17] Bass A.J., Thorsson V., Shmulevich I., Reynolds S.M., Miller M., Bernard B., Hinoue T., Laird P.W., Curtis C., Shen H. (2014). Comprehensive molecular characterization of gastric adenocarcinoma. Nature.

[18] Wang Y.-L., Lee C.-C., Shen Y.-C., Lin P.-L., Wu W.-R., Lin Y.-Z., Cheng W.-C., Chang H., Hung Y., Cho Y.-C. (2021). Evading immune surveillance via tyrosine phosphorylation of nuclear PCNA. Cell Rep..

[19] Duursma A.M., Driscoll R., Elias J.E., Cimprich K.A. (2013). A role for the MRN complex in ATR activation via TOPBP1 recruitment. Mol. Cell.

[20] Clarke M.F., Dick J.E., Dirks P.B., Eaves C.J., Jamieson C.H., Jones D.L., Visvader J., Weissman I.L., Wahl G.M. (2006). Cancer stem cells--perspectives on current status and future directions: AACR Workshop on cancer stem cells. Cancer Res..

[21] Ginestier C., Hur M.H., Charafe-Jauffret E., Monville F., Dutcher J., Brown M., Jacquemier J., Viens P., Kleer C.G., Liu S. (2007). ALDH1 is a marker of normal and malignant human mammary stem cells and a predictor of poor clinical outcome. Cell Stem Cell.

[22] Dontu G., Al-Hajj M., Abdallah W.M., Clarke M.F., Wicha M.S. (2003). Stem cells in normal breast development and breast cancer. Cell Prolif..

[23] Shaw F.L., Harrison H., Spence K., Ablett M.P., Simões B.M., Farnie G., Clarke R.B. (2012). A Detailed Mammosphere Assay Protocol for the Quantification of Breast Stem Cell Activity. J. Mammary Gland. Biol. Neoplasia.

[24] Sancho P., Burgos-Ramos E., Tavera A., Bou Kheir T., Jagust P., Schoenhals M., Barneda D., Sellers K., Campos-Olivas R., Graña O. (2015). MYC/PGC-1α Balance Determines the Metabolic Phenotype and Plasticity of Pancreatic Cancer Stem Cells. Cell Metab..

[25] Sancho P., Barneda D., Heeschen C. (2016). Hallmarks of cancer stem cell metabolism. Br. J. Cancer.

[26] Peng B., Ortega J., Gu L., Chang Z., Li G.M. (2019). Phosphorylation of proliferating cell nuclear antigen promotes cancer progression by activating the ATM/AKT/GSK3beta/Snail signaling pathway. J. Biol. Chem..

[27] Sahai E., Astsaturov I., Cukierman E., DeNardo D.G., Egeblad M., Evans R.M., Fearon D., Greten F.R., Hingorani S.R., Hunter T. (2020). A framework for advancing our understanding of cancer-associated fibroblasts. Nat. Rev. Cancer.

[28] Huang T.X., Guan X.Y., Fu L. (2019). Therapeutic targeting of the crosstalk between cancer-associated fibroblasts and cancer stem cells. Am. J. Cancer Res..

[29] Iwamoto H., Izumi K., Mizokami A. (2020). Is the C-C Motif Ligand 2–C-C Chemokine Receptor 2 Axis a Promising Target for Cancer Therapy and Diagnosis?. Int. J. Mol. Sci..

[30] Wang X., Spandidos A., Wang H., Seed B. (2012). PrimerBank: A PCR primer database for quantitative gene expression analysis, 2012 update. Nucleic Acids Res..

